# Epidemiological characteristics and transmission dynamics of the early stage Chikungunya fever outbreak in Foshan City, Guangdong Province, China in 2025

**DOI:** 10.1186/s40249-025-01364-y

**Published:** 2025-09-11

**Authors:** Meng Zhang, Yihong Li, Xiqing Huang, Man Liu, Siyang Jiang, Biao Zeng, Luxiang Ouyang, Jianhua Huang, Bing Mai, Qihua Guan, Jiazhi Zeng, Muying Fu, Bingu Zhuo, Yawen Liu, Qin Zeng, Naling Zhu, Tao Wang, Xiaojun Huang, Yimin Pan, Mingji Cheng, Penghui Jia, Xiaofang Peng, Jinhua Duan, Baisheng Li, Jianfeng He, Yanping Zhang, Lei Zhou, Min Kang, Jianpeng Xiao, Zefeng Yang, Yan Li

**Affiliations:** 1 Guangdong Provincial Center for Disease Control and Prevention, Guangzhou, Guangdong Province China; 2 Foshan Center for Disease Control and Prevention, Foshan, Guangdong Province China; 3 Guangdong Provincial Institute of Public Health, Guangzhou, Guangdong Province China; 4 Chinese Field Epidemiology Training Program, Beijing, China; 5 Hubei Provincial Center for Disease Control and Prevention, Wuhan, Hubei China; 6 School of Public Health, Sun Yat-Sen University, Guangzhou, Guangdong Province China; 7 Guangdong Provincial Field Epidemiology Training Program, Guangzhou, Guangdong Province China; 8 Dongguan Center for Disease Control and Prevention, Dongguan, Guangdong Province China; 9 Yantian District Center for Disease Control and Prevention, Shenzhen, Guangdong Province China; 10 Shanwei Center for Disease Control and Prevention, Shanwei, Guangdong Province China; 11 Tianhe District Center for Disease Control and Prevention, Guangzhou, Guangdong Province China; 12 Yuexiu District Center for Disease Control and Prevention, Guangzhou, Guangdong Province China; 13 Maoming Center for Disease Control and Prevention, Maoming, Guangdong Province China; 14 Liwan District Center for Disease Control and Prevention, Guangzhou, Guangdong Province China; 15 Chinese Center for Disease Control and Prevention, Beijing, China

**Keywords:** Chikungunya fever, Outbreak, Epidemiology, Transmission dynamics, China

## Abstract

**Background:**

As of July 22, 2025, the chikungunya virus transmission has been documented across 119 countries and territories of the world. In 2025, an outbreak of chikungunya fever (CF) occurred in Foshan, Guangdong Province, China. We aimed to analyze the epidemiological characteristics and transmission dynamics during the early stage of this outbreak.

**Methods:**

We collected the data of CF cases in Foshan from July 8 to July 26, 2025. Case data were extracted from the National Notifiable Infectious Disease Reporting System. Demographics and tempo-spatial distributions of cases, incidence rates and the onset-to-report interval times were analyzed. Global spatial autocorrelation (Moran’s *I*) to assess township-level clustering; Kruskal–Wallis tests with Dunn's post-hoc comparisons (Bonferroni-corrected) to analyze onset-to-report intervals across four epidemic phases. The basic reproduction number (*R*_0_) was calculated using a maximum likelihood method, which was also compared with the *R*_0_ from the CF outbreak in Dongguan City of Guangdong Province in 2010.

**Results:**

A total of 4,754 local cases were reported during the study period. Persons aged 65 years or above had the highest incidence (116.57 per 100,000 population). Most cases were business/service workers, homemakers, and retirees. The median onset-to-report interval decreased from 4 days to 1 day after outbreak control measures were implemented. The outbreak, initially detected in Shunde District, spread rapidly to other districts of Foshan, forming a significant spatial cluster (Moran's *I* = 0.152, *P* = 0.029). The estimated *R*_0_ was 16.3 (95% confidence interval: 15.0 to 17.5), substantially higher than the estimated *R*_0_ of 5.5 for the Dongguan outbreak in 2010.

**Conclusions:**

This outbreak was characterized by high transmissibility, with older persons being a primary high-risk group. The rapid reduction in case reporting delay highlights the effectiveness of response interventions. Sustained, integrated and prompt response has been essential to control the outbreak.

**Graphical Abstract:**

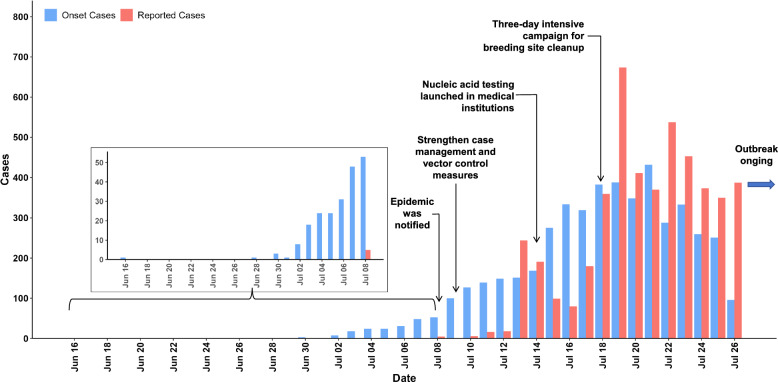

**Supplementary Information:**

The online version contains supplementary material available at 10.1186/s40249-025-01364-y.

## Background

Chikungunya fever (CF) is an arboviral disease caused by infection with the chikungunya virus (CHIKV). CHIKV is an enveloped, positive-sense, single-stranded RNA virus in the *Togaviridae* family, *Alphavirus* genus [1], and transmitted through the bite of infected *Aedes* spp. Mosquitoes [2]. CHIKV infection typically causes an acute febrile illness with severe arthralgia affecting multiple joints, which can persist for months or longer in more than 30% of patients [3]. Although the overall mortality of CHIKV infection is low, severe manifestations can occur, particularly in infants, the elderly, and patients with underlying medical conditions [4]. CHIKV was first identified in Tanzania in 1952 [5]. It became a global public health issue during the 2004 Kenya outbreak when it spread rapidly across the Indian Ocean islands and affected millions of people [5]. In recent years, the world has seen an accelerated expansion of the disease in numerous countries and areas, including a large-scale outbreak in Réunion in nearly two decades, and with ongoing transmissions in Brazil, India, Sri Lanka, etc. [6]. In 2025, as of early June, approximately 220,000 cases and 80 related deaths have been reported across 14 countries in the Americas, Africa, Europe and Asia [7]. It is estimated that over three-quarters of the world’s population lives in areas at risk of CHIKV transmission, and to date, no efficacious medical countermeasures are available [5]. In July 2025, the World Health Organization (WHO) issued a global risk warning underscoring the heightened international concern [8].

In China, CF outbreaks have primarily occurred after imported cases, with occasional sporadic local transmission identified in Guangdong, Zhejiang, and Yunnan provinces [9–11]. Notably, Guangdong Province documented two locally transmitted outbreaks in 2010 and 2019, respectively. In 2010, Dongguan City reported the first CF outbreak in China with 282 confirmed cases, which was triggered by an imported case [9, 12]. Another outbreak occurred in a rural area of Beijiao Township, Shunde District, Foshan in 2019, involving 4 confirmed cases. Between 2010 and 2024, CF cases in China were primarily dominated by imported cases and occasional sporadic transmission. The subtropical climate of Guangdong Province, favors widespread distribution of *Aedes albopictus*, the primary mosquito vector for both dengue and CF, making the province a high-risk area for arboviral diseases [13].

In July 2025, an unexpected large outbreak of CF was identified in Foshan City, Guangdong Province, China. As of August 15, 2025, the outbreak is still ongoing. In this study, we aim to address the epidemiological characteristics, evaluate the case detection efficiency, and estimate the transmission dynamics during the early stage of this outbreak.

## Methods

### Study site

Foshan, located at 22°38'N–23°34'N, and 112°22'E–113°23'E, is a major city situated in central-southern Guangdong Province of China, which has historically been burdened by arboviral diseases. The city’s subtropical monsoon climate, with a rainy season spanning from April to September, creates a favorable environment for the proliferation of the *Aedes* spp. Mosquitoes [14]. As a core urban center in the densely populated Pearl River Delta (PRD), Foshan is characterized by both a high population density (2532 persons/km^2^ in 2023) and extensive international trade, with an annual import–export volume exceeding CNY 5.9 trillion [15]. The city faces a perennial threat of mosquito-borne disease outbreaks; for instance, the first documented dengue epidemic in Guangdong Province occurred in Foshan in 1978 [16].

### Data sources

Case data were extracted from the National Notifiable Infectious Disease Reporting System (NNDRS). The NNDRS is a real-time, internet-based surveillance system covering the Chinese mainland, and its operational protocols have been described in previous studies [17, 18]. All medical institutions are mandated to report all suspected, probable, and confirmed CF cases to the NNDRS within 24 h of diagnosis, according to the CF prevention and control protocol (2025 Edition) [19]. All cases included in this analysis were laboratory-confirmed via reverse transcription polymerase chain reaction (RT-PCR) [20].

CF case data in Foshan from July 8 to July 26, 2025, and historical dengue fever case data in Guangzhou (2014), Chaozhou (2015), and Shantou (2019) were obtained. The individual case records used in this study were anonymized and only included demographic details (gender, age), residential address, occupation, and the dates of symptom onset and case reporting. The identity of individual cases could not be uncovered. This disease surveillance activity was determined by the National Health Commission of the People’s Republic of China to be part of routine public health surveillance, and thus exempted from institutional ethical review board assessment [21]. To estimate the incidence rate, the population data of Foshan, stratified by gender, age group, and district, were also obtained from the NNDRS.

### Statistical analysis

#### Epidemiological patterns of the outbreak

We analyzed the epidemiological characteristics of the early stage of this outbreak by examining the demographic and tempo-spatial distributions of reported cases. The epidemiological features of local cases were summarized by gender, age and occupation, and corresponding crude incidence rates were calculated. For the temporal analysis, we constructed epidemic curves according to symptom onset date and case reporting date respectively to visualize the outbreak’s progression. The spatial distribution was explored using phased, township-level geospatial mapping to illustrate the geographic spread of cases during distinct periods of the epidemic. To further explore the clustering of cases at the township level, we conducted a global spatial autocorrelation analysis on the case distribution as of July 26, and the Moran’s *I* was calculated.

The study period was divided into four phases (July 8–12, July 13–17, July 18–22, July 23–26) to facilitate trend analysis. To assess and visualize case detection efficiency across these phases, we analyzed the onset-to-report interval, displaying the distributions using box plots. Accordingly, data were summarized using the median and interquartile range (IQR). A Shapiro–Wilk test was first performed to assess the normality of the interval data within each phase. The Kruskal–Wallis test was used for the overall comparison across the four phases, followed by Dunn’s post-hoc test with Bonferroni correction for pairwise comparisons.

#### Estimation of the basic reproduction number (*R*_0_)

Based on the data from the early stage of the CF outbreak in Foshan, with illness onset dates ranging from June 16 to July 15, the basic reproduction number (*R*_*0*_) and its 95% confidence interval were estimated. The *R*_0_ was calculated using a maximum likelihood method based on a gamma-distributed generation time (GT) of 14 days [standard deviation (*SD*): 6.4 days] [22–24]. *R*_0_ was defined as the mean number of secondary cases a single infectious person causes during the generation time in a completely susceptible population.

To study the differences in transmissibility between this outbreak and previous ones, we further estimated the transmissibility of several previous arthropod-borne infectious disease outbreaks in Guangdong. We first calculated the *R*_*0*_ for the outbreak of CF in Dongguan City in 2010 [9]. Then, we estimated the *R*_0_ of three major dengue outbreaks (Guangzhou in 2014, Chaozhou in 2015, Shantou in 2019) in Guangdong Province [25–27]. To further compare the transmission rate, the exponential growth rate and the doubling time were calculated. The time series data for these previous outbreaks were obtained from the Guangdong Provincial Center for Disease Control and Prevention (Figure S1).

#### Sensitivity analysis

To assess the robustness of the *R*_0_ estimates, we conducted a sensitivity analysis. First, we re-estimated *R*_0_ using the exponential growth method [28]. Second, we varied the time window for the maximum Likelihood estimation by shifting the start and end dates of the case series, using periods from June 28 to July 15 and from June 28 to July 16 for re-calculation.

#### Analysis software

R software (version 4.2.0; R Foundation for Statistical Computing, Vienna, Austria) was used for data analysis and plotting. The “spdep” package was used for spatial autocorrelation analysis, and the “*R*_0_” package was used for calculating *R*_0_ [28]. Two-tailed *P* < 0.05 was considered statistically significant for all statistical tests.

## Results

### Demographic distribution

During the study period from July 8 to July 26, 2025, a total of 4754 CF cases were reported in Foshan, which were all local cases, corresponding to an overall crude incidence rate of 49.44 per 100,000 population. The median age of patients was 44 (IQR: 27–60) years, with a male-to-female ratio of 1.04: 1 [95% confidence interval (*CI*) 0.97–1.09]. All reported cases were clinically mild, with no severe cases or deaths recorded. The demographic distribution revealed that individuals aged 65 years or above had the highest incidence rate (116.57 per 100,000 population) among all age groups. Regarding occupational status, cases were predominantly concentrated among business/service workers (36.90%), followed by homemakers/unemployed individuals (25.52%) and retirees (14.20%). Together, these three groups made up 76.62% of all reported cases (Table 1).

**Table 1:** Incidence and proportional distribution of Chikungunya fever cases in Foshan, China, by gender, age group, and employment status (July 8–July 26, 2025)

Variable	Shunde district		Other districts in Foshan		Overall in Foshan	
	Cases (incidence rate /10^5^)	*P* value	Cases (incidence rate /10^5^)	*P* value	Cases (incidence rate /10^5^)	*P* value
Total cases	4208 (128.81)		546 (8.60)		4754 (49.44)	
Gender						
Male	2134 (120.52)	< 0.001	285 (8.33)	0.419	2419 (46.58)	< 0.001
Female	2074 (138.63)		261 (8.92)		2335 (52.81)	
Male-to-female ratio	1.03		1.09		1.04	
Age group (years)						
< 5	97 (77.33)	< 0.001	12 (4.47)	< 0.001	109 (27.67)	< 0.001
5−14	481 (138.46)		58 (8.50)		539 (52.33)	
15−24	349 (114.41)		48 (8.41)		397 (45.34)	
25−34	544 (87.31)		63 (5.18)		607 (32.99)	
35−44	641 (93.35)		85 (6.53)		726 (36.50)	
45−54	661 (113.64)		80 (7.33)		741 (44.28)	
55−64	634 (186.10)		68 (10.12)		702 (69.34)	
≥ 65	801 (312.00)		132 (24.28)		933 (116.57)	

	*N* (%)		*N* (%)		*N* (%)	
Employment status						
Business/service workers	1595 (37.90%)	< 0.001	159 (29.12%)	< 0.001	1754 (36.90%)	< 0.001
Homemakers/unemployed	1120 (26.62%)		93 (17.03%)		1213 (25.52%)	
Retirees	568 (13.50%)		107 (19.60%)		675 (14.20%)	
Students	458 (10.88%)		56 (10.26%)		514 (10.81%)	
General staff/workers	194 (4.61%)		37 (6.78%)		231 (4.86%)	
Nursery children	90 (2.14%)		17 (3.11%)		107 (2.25%)	
Scattered children	82 (1.95%)		9 (1.65%)		91 (1.91%)	
Farmers	62 (1.47%)		23 (4.21%)		85 (1.79%)	
Others	39(0.93%)		45 (8.75%)		84 (1.77%)	

### Time from onset to reporting

**Fig. 1:**
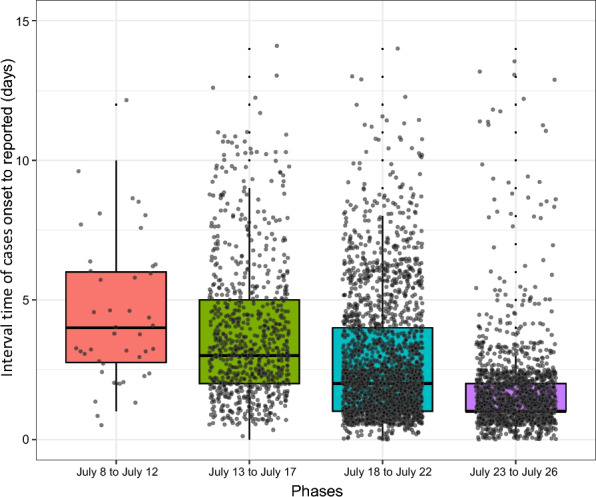
Changes of interval time of the onset-to-report during different phases in the early stage of the chikungunya fever outbreak in Foshan, 2025

The interval from symptom onset to case report showed a statistically significant decreasing trend throughout the study period (Kruskal–Wallis test, *χ*^2^ = 810.8, *P* < 0.001). Specifically, the median onset-to-report interval decreased from 4 days (IQR: 3–6) during the initial phase of the epidemic (July 8–12) to 1 day (IQR: 1–2) during a later phase (July 23–26**)** (Fig. 1). Dunn’s post-hoc test with Bonferroni correction revealed that, with the exception of the comparison between the first (July 8–12) and second (July 13–17) phases (*P* = 0.881), all other pairwise comparisons showed highly significant differences (*P* < 0.001). Comprehensive descriptive statistics for the onset-to-report interval during each phase are provided in Table S1.

### Temporal distribution

**Fig. 2:**
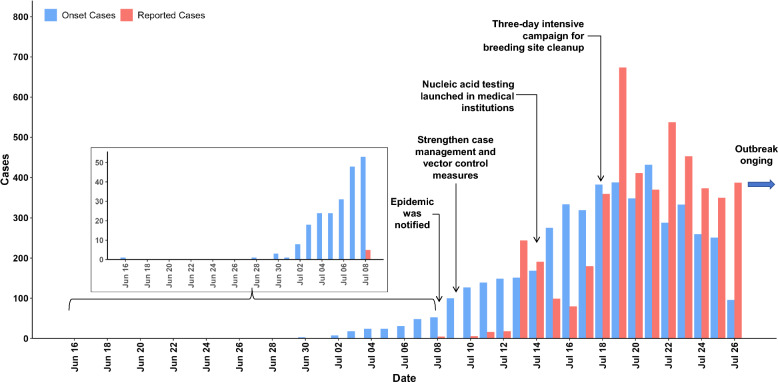
Distribution of chikungunya fever cases by symptom onset date and reporting date in Foshan, between July 8 and July 26 in 2025

Epidemiological investigation revealed that the index case of this outbreak, identified in Shunde District of Foshan, was laboratory-confirmed on July 8. Localized clustered transmission patterns were demonstrated initially, followed by rapid geographical expansion with cases subsequently reported in other districts of Foshan from July 11 onwards. Epidemic curves according to symptom onset date and case reporting date respectively are presented in Fig. 2. Number of cases based on symptom onset date peaked at 432 cases on July 21 (82.6% from Shunde District). The number of cases based on reporting date peaked at 674 cases on July 19 (93.6% from Shunde District). The CF epidemic was notified on July 8. Subsequently, a series of interventions was implemented in Foshan. These included the launch of nucleic acid testing in medical institutions on July 14, followed by a three-day intensive campaign for clearance of mosquito breeding sites from July 18 to July 20.

### Spatial distribution

**Fig. 3:**
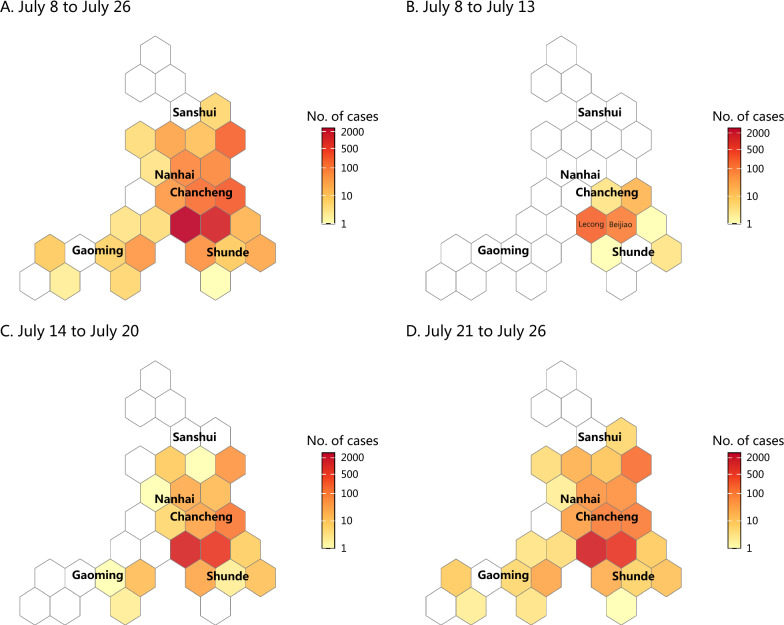
Phased geographic distribution of chikungunya fever cases in Foshan between July 8 and July 26, 2025 (This map shown is not a true boundary map of Foshan; it has been converted into a hexagonal map)

The spatiotemporal analysis demonstrated a highly clustered outbreak pattern, with Shunde District serving as the epicenter, accounting for 89% of all reported cases (Fig. 3). Initially (July 8–13), cases were concentrated in localized hotspots within the towns of Lecong and Beijiao in Shunde District. The outbreak subsequently expanded geographically, spreading to the adjacent districts of Chancheng and Nanhai by July 20. Following this, sporadic transmission was observed in the more distant Gaoming and Sanshui districts, reflecting a classic pattern of radial dissemination from a primary focus. The global spatial autocorrelation analysis confirmed significant positive spatial autocorrelation of cases at the township level (Moran’s *I* = 0.152, *P* = 0.029). This indicates that the case distribution was spatially clustered.

### Transmission dynamics

The basic reproduction number (*R*_0_) was estimated to be 16.3 (95% *CI* 15.0−17.5) for this CF outbreak in Foshan. The corresponding exponential growth rate (r) was approximately 0.2 per day, with an epidemic doubling time (T_d_) of 3.5 days. From the result of transmission dynamics for other major arbovirus outbreaks in Guangdong Province (Table 2), we found that the *R*_0_ estimated for CF outbreak in Dongguan City in 2010 is 5.5 (95% *CI* 4.2−7.1). The *R*_0_ of CF outbreak in Foshan is also higher than those of the major dengue outbreaks in Guangzhou City in 2014 (*R*_0_ = 11.6), Chaozhou City in 2015 (*R*_0_ = 14.1), and Shantou City in 2019 (*R*_0_ = 12.2).

**Table 2:** Comparison of transmission dynamics of outbreaks between chikungunya fever, and dengue in Guangdong Province

Outbreak events	Basic reproduction number (*R*_0_, 95% *CI*)	Exponential growth rate (per day)	Doubling time (days)
Chikungunya fever in Foshan, 2025	16.3 (15.0−17.5)	0.20	3.5
Chikungunya fever in Dongguan city, 2010	5.5 (4.2−7.1)	0.12	5.7
Dengue in Guangzhou city, 2014	11.6 (8.6−15.3)	0.18	4.0
Dengue in Chaozhou city, 2015	14.1 (11.5−17.0)	0.19	3.7
Dengue in Shantou city, 2019	12.2 (8.3−17.1)	0.18	3.9

The *R*_0_ estimated for chikungunya fever and dengue based on the same generation time of 14.0 days [24]. *CI* Confidence interval

From the result of the sensitivity analysis, a comparable *R*_0_ of 15.3 (95% *CI* 13.7−17.2) was obtained using the exponential growth method. Shifting the estimation window to later periods (June 28–July 15 and June 28–July 16) also produced consistent *R*_0_ estimates of 16.4 (95% *CI*: 15.2−17.7) and 14.9 (95% *CI* 13.9−15.9), respectively. These results confirmed the robustness of our primary *R*_0_ estimate.

## Discussion

Based on data from the early stage of a large CF outbreak in Foshan in 2025, we described the epidemiological characteristics and transmission dynamics. For the CF outbreak in Foshan, we estimated a basic reproduction number (*R*_0_) of 16.3 (95% *CI* 15.0−17.5). This high value indicated that CF is highly transmissible via mosquitoes, with a single infected individual would, on average, transmit the virus to more than 16 secondary cases over 14 days of a generation time. Intensified and sustained vector control is needed to contain the current outbreak. Meanwhile, a robust surveillance system should be in place to detect future outbreaks and control them as early as possible.

None of the cases in our study have reported any history of international travel. Significant local transmission of the disease resulted in a cumulative total of 4754 confirmed cases by July 26, 2025, with the majority concentrated in Shunde District of Foshan. The scale of this outbreak has far exceeded the outbreak of Dongguan City in 2010, making it the largest CF outbreak documented in China to date. It occurred alongside a notable global resurgence of the virus, including an ongoing outbreak on Réunion with over 53,000 cases reported as of early 2025 [29]. Frequent international travels may increase the risk of imported cases that lead to local CF outbreaks in internationally connected cities such as Foshan. Shunde District of Foshan, the epicenter of the outbreak, has had long-standing socioeconomic ties with Réunion and is home to a large diaspora community from the island [30]. While a definitive importation link cannot be established from our study, such known connection between Foshan and Reunion represents a plausible channel for introduction of the virus. The genomic sequencing data from our investigation, which identified the virus as the East-Central-South African (ECSA) genotype prevalent in the Indian Ocean region, further supported the hypothesis of an importation event from an endemic area [20].

The rapid escalation of the outbreak has likely been fueled by highly favorable local environmental factors and delays in identifying initial cases. Foshan’s subtropical monsoon climate is suitable for *Aedes* vectors. This risk was significantly amplified by an intense precipitation event in mid-to-late June (June 12–20), where rainfall surged 130% above the seasonal norm, creating small water bodies and favorable conditions for the proliferation of mosquito breeding sites [14, 31]. Furthermore, higher temperatures can significantly shorten the extrinsic incubation period of CHIKV in the mosquito vector, thereby dramatically accelerating transmission efficiency [32]. The situation was compounded by the fact that there was a 22-day interval between the symptom onset of the first probable case and its reporting. There may have been intense human-mosquito-human transmission within a dense, immunologically naive urban population during this gap period before response interventions could be fully implemented. These findings underscore the imperative to intensify surveillance and early warning systems for mosquito-borne diseases, so that cases can be detected early and outbreaks swiftly contained.

The current study found that older persons aged 65 years or above had the highest incidence. Such finding is consistent with observations from other non-endemic areas where infection rate increases with age [33], but contrasts with reports from endemic countries like India and Colombia where children are predominantly affected [34, 35]. This discrepancy may reflect differences in population immunity. In an immunologically naive population in Foshan, intrinsic vulnerabilities such as age-related comorbidities are risk factors for severe outcomes and primary determinants of infection risk [36]. In endemic regions, acquired immunity among adults shifts the burden of disease to younger, susceptible age groups. Apart from age, occupation can be a major risk factor. The higher risk among business and service workers, consistent with findings from a study in Guangzhou [37], is likely attributable to high mobility and prolonged exposure to densely populated commercial areas. Similarly, the high proportion of cases among homemakers, unemployed individuals, and retirees, a pattern also observed in New Delhi [38], highlights the significance of peridomestic transmission, as these groups tend to spend more time at or near their homes during the daytime peak biting activity of *Aedes* mosquitoes. These findings suggest that prevention strategies should be tailored to the specific exposure risks of different demographic and occupational groups.

The interval between symptom onset and reporting decreased significantly as the outbreak progressed, from a median of 4 days to 1 day. This rapid improvement can be attributed to several synergistic and multi-pronged public health measures. First, the city-wide implementation of a synchronous screening strategy for dengue and CF in patients presenting with fever, rash, or arthralgia improved early detection capability. This strategy was enhanced by the broad rollout of CHIKV nucleic acid testing across all secondary and tertiary medical institutions from July 14, which substantially accelerated case confirmation. In parallel, targeted trainings were conducted to equip primary healthcare physicians to better identify potential cases and facilitate timely referrals. Extensive community-based health promotion campaigns increased awareness and encouraged prompt care-seeking behavior among residents.

The model yielded an estimated *R*_0_ of 16.3 for the CF outbreak, a value indicating exceptionally high transmissibility. This *R*_0_ far exceeds the *R*_0_ for the CF outbreak in Dongguan City (5.5). It surpasses estimates from the 2017 Central Italy outbreak (*R*_0_ = 2.1) [23] and the global mean (*R*_0_≈3.4) [39], but is slightly below the *R*_0_ of 20.1 reported from the 1997–1998 Malaysia outbreak [40]. When compared to the COVID-19 Delta variant outbreak in Guangzhou City in 2021 [41], although the *R*_0_ was considerably higher (16.3 vs 3.2), the exponential growth rate of the CF outbreak was lower (0.2 per day vs 0.4 per day) due to a different generation time. Nevertheless, this great transmissibility aligns with the local context of a subtropical climate, rural–urban fringe areas with poorer sanitation, abundant mosquito breeding sites, and high population density, all of which are conditions that favor amplifying vector abundance and virus circulation. Accordingly, the high transmission dynamic of the outbreak highlights an urgent need for rigorous public-health measures, such as enhanced case surveillance and management systems, integrated vector barrier interventions for confirmed case management, and community-wide vector control operations [42].

The primary strength of this study is its characterization of the epidemiological features and initial transmission dynamics during the early stage of the CF outbreak. The timely estimation of the basic reproduction number provides important insights for assessing the transmission risk and tailoring public health responses. However, this study is subject to several limitations. First, the data were derived from a passive public health surveillance system, which is inherently susceptible to underreporting resulting from variations in reporting practices, healthcare accessibility, and laboratory capacity. While extensive community engagement may have enhanced case finding, the inherent limitations of such a reporting system likely mean the true scale of the outbreak was underestimated. Second, the potential for case underreporting between June and July, as well as uncertainty regarding the index case identification, may have led to upward bias for *R*_0_. Nonetheless, sensitivity analyses yielded consistent *R*_0_ estimates using exponential growth models, and using shifted time windows (June 28–July 15/16). Third, as the individual case records used in this study were anonymized and contained no personally identifiable information, retrospective household clustering analyses could not be conducted. Fourth, the use of passive surveillance data precluded linkage to detailed clinical records, including symptom profiles and disease progression. We were therefore unable to conduct a comprehensive analysis of clinical features or clinical-epidemiological correlations. Future investigations integrating detailed clinical data were required to address these knowledge gaps.

## Conclusions

The CF outbreak in Foshan in 2025 was characterized by high transmissibility and identified older persons as a high-risk group. Intensive public health interventions contributed to a swift reduction in the delay between symptom onset and case reporting. Enhanced case surveillance and management systems, integrated vector barrier interventions, community engagement, and territory-wide vector control operations are crucial in reducing the risk of future large-scale outbreaks in similar subtropical and internationally connected regions.

## Supplementary Information

Supplementary material 1. Table S1. Descriptive statistics of the onset-to-report interval (days) during the early phases of the 2025 Foshan chikungunya fever outbreak.

Supplementary material 2. Figure S1. Epidemic curves of the 2010 chikungunya fever outbreak in Dongguan City and three dengue outbreaks in Guangdong Province.

## Data Availability

The datasets used and/or analyzed during the current study are available from the corresponding author on reasonable request.

## Abbreviations

CF: Chikungunya fever
CHIKV: Chikungunya virus
*CI*: Confidence interval
COVID-19: Coronavirus disease 2019
ESSA: East-Central-South African
GT: Generation time
NNDRS: National notifiable infectious disease reporting system
PRD: Pearl river delta
*R*_0_: Basic reproduction number
RT-PCR: Reverse transcription polymerase chain reaction
*SD*: Standard deviation
T_d_: Doubling time
WHO: World Health Organization
