# Supplementary material for: Epidemiological characteristics and transmission dynamics of the early stage Chikungunya fever outbreak in Foshan City, Guangdong Province, China in 2025
Source: Infect Dis Poverty. 2025 Sep 11;14:93. doi: 10.1186/s40249-025-01364-y (PMC12424219; doi:10.1186/s40249-025-01364-y)
Supplement: Supplementary file 1 — Supplementary material 1. Table S1. Descriptive statistics of the onset-to-report interval (days) during the early phases of the 2025 Foshan chikungunya fever outbreak. [file 40249_2025_1364_MOESM1_ESM.docx]

[**Additional file 1** **Table S1**.](https://static-content.springer.com/esm/art:10.1186/s40249-024-01189-1/MediaObjects/40249_2024_1189_MOESM1_ESM.pdf) Descriptive statistics of the onset-to-report interval (days) during the early phases of the 2025 Foshan Chikungunya fever outbreak

| **Period** | **Mean** | **SD** | **25th percentile (Q1)** | **Median** | **75th percentile (Q3)** |
| --- | --- | --- | --- | --- | --- |
| July 8–12 | 4.91 | 3.94 | 3 | 4 | 6 |
| July 13–17 | 3.87 | 2.53 | 2 | 3 | 5 |
| July 18–22 | 2.72 | 2.13 | 1 | 2 | 4 |
| July 23–26 | 1.61 | 1.58 | 1 | 1 | 2 |
