# Supplementary figures and images for: Epidemiological characteristics and transmission dynamics of the early stage Chikungunya fever outbreak in Foshan City, Guangdong Province, China in 2025
Source: Infect Dis Poverty. 2025 Sep 11;14:93. doi: 10.1186/s40249-025-01364-y (PMC12424219; doi:10.1186/s40249-025-01364-y)

**Chikungunya in Dongguan, 2010**

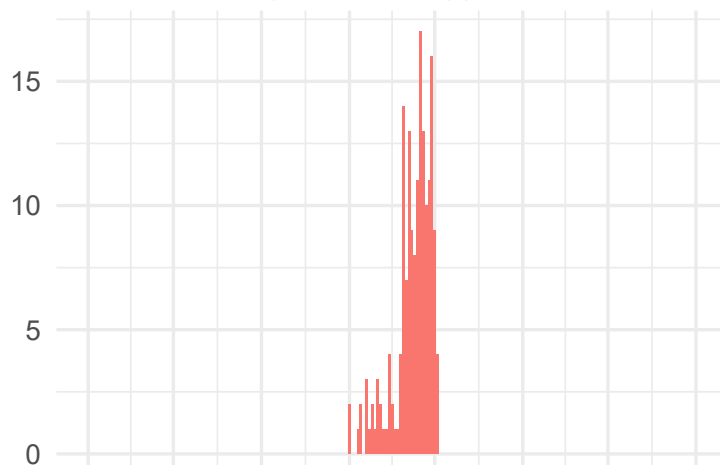

**Dengue in Guangzhou, 2014**

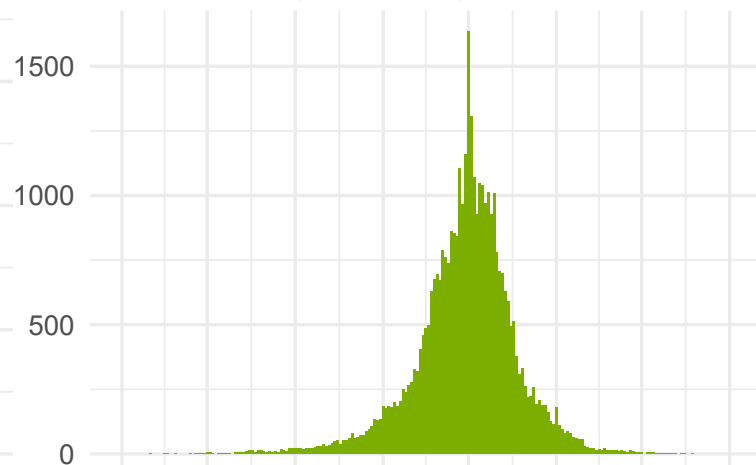

**Dengue in Chaozhou, 2015**

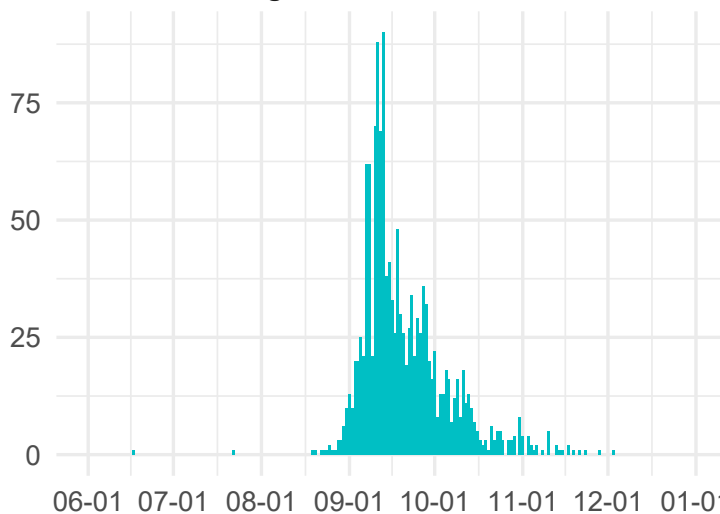

**Dengue in Shantou, 2019**

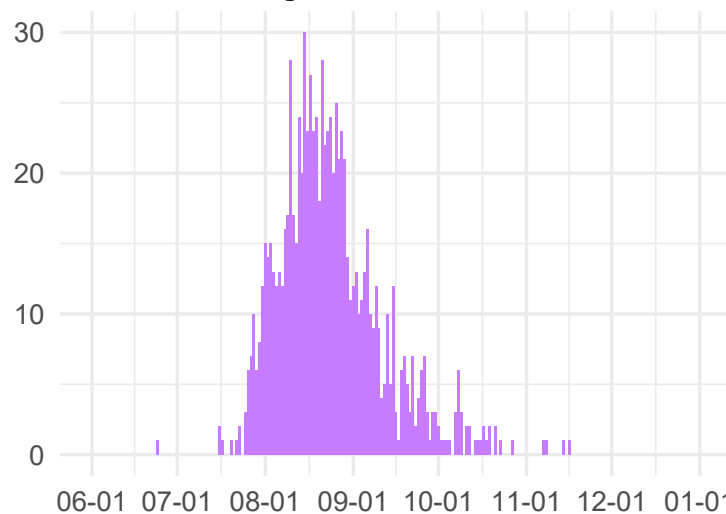

Date

Supplement: Supplementary file 2 — Supplementary material 2. Figure S1. Epidemic curves of the 2010 chikungunya fever outbreak in Dongguan City and three dengue outbreaks in Guangdong Province. [file 40249_2025_1364_MOESM2_ESM.pdf]
